# Supplementary material for: Comprehensive multi-omics integration identifies differentially active enhancers during human brain development with clinical relevance
Source: Genome Med. 2021 Oct 19;13:162. doi: 10.1186/s13073-021-00980-1 (PMC8524963; doi:10.1186/s13073-021-00980-1)
Supplement: Supplementary file 1 — Additional file 1: Fig. S1: Flow chart of integrative data analysis; Fig. S2: Derivation of pCRs and DAEs; Fig. S3: Enhancer-Gene predictions and target gene expression; Fig. S4: Features and motifs in DAEs and nDAEs; Fig. S5: Cell type specificity of DAEs and nDAEs; Fig. S6: Dynamics of DAEs and nDAEs in comparison to adult brain; Fig. S7: DAEs and nDAEs in human disease, related to Fig. 5; Fig. S8: Zebrafish enhancer reporter assay [file 13073_2021_980_MOESM1_ESM.pdf]

## Additional File 1

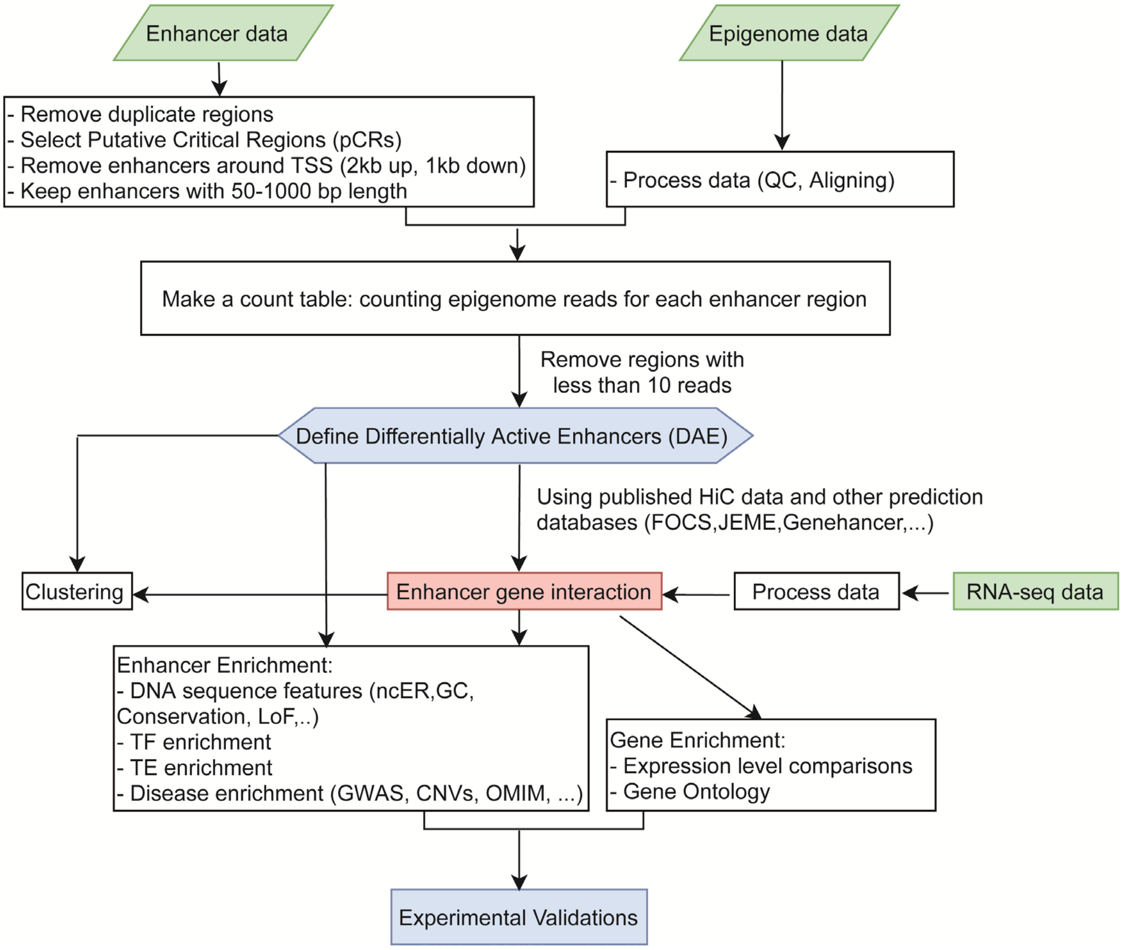


**Fig. S1:** ***Flow chart of integrative data analysis***

Overview of the various analysis steps performed in this study. See text and methods for additional details.

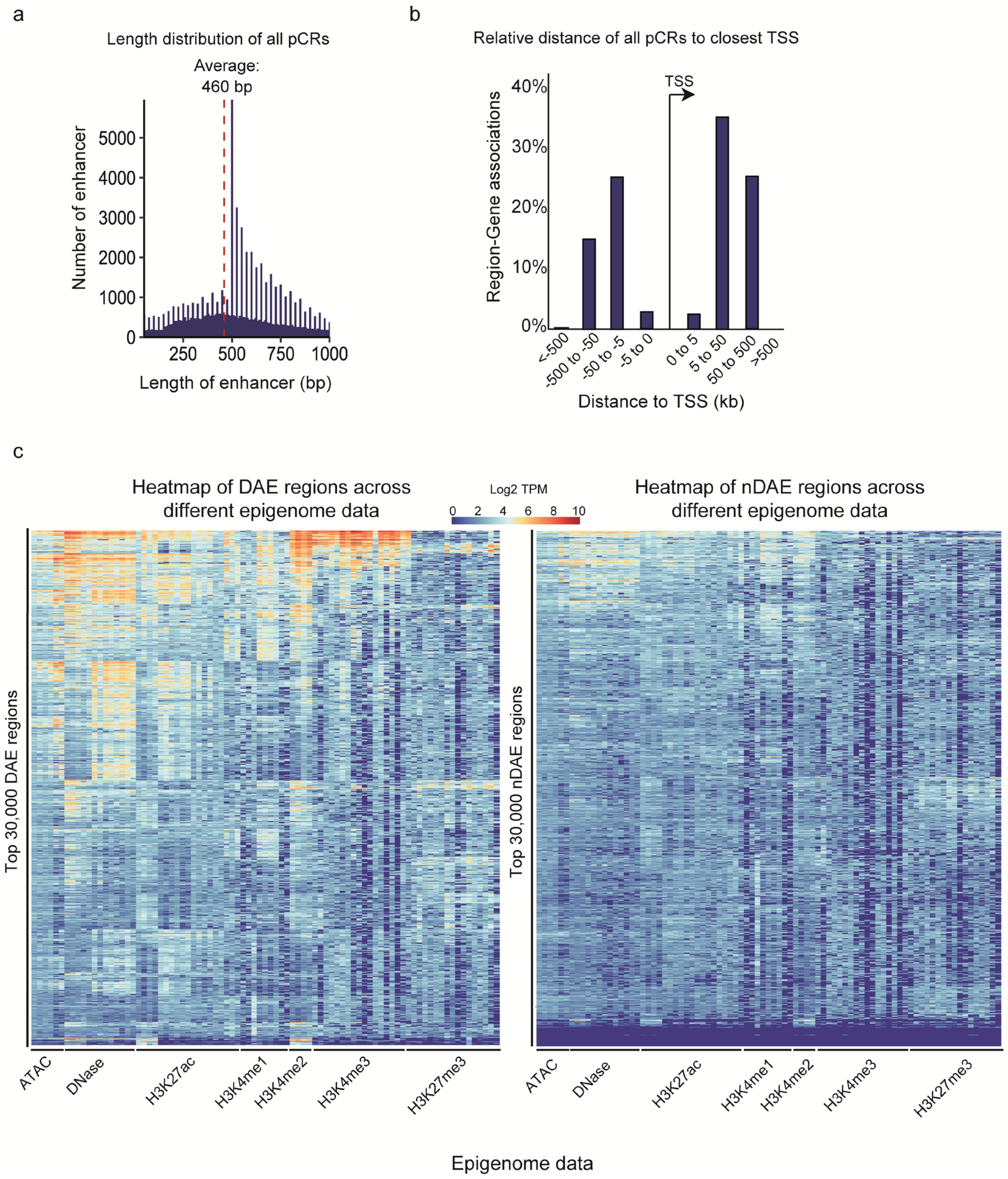


**Fig. S2: *Derivation of pCRs and DAEs***

A) Density plot showing the size distribution of the 202,163 pCRs in bps. The red dashed line indicates the average length of all pCRs (460 bp).

B) Relative distribution of all 202,163 pCRs in relation to their closest transcriptional start site. Graph generated using GREAT [1].

C) Heatmaps showing variability across all epigenome features for the top 30,000 DAEs (left) and nDAEs (right). Columns represent in total 494 epigenome data sets used for the various types of histone marks and chromatin accessibility, as indicated.

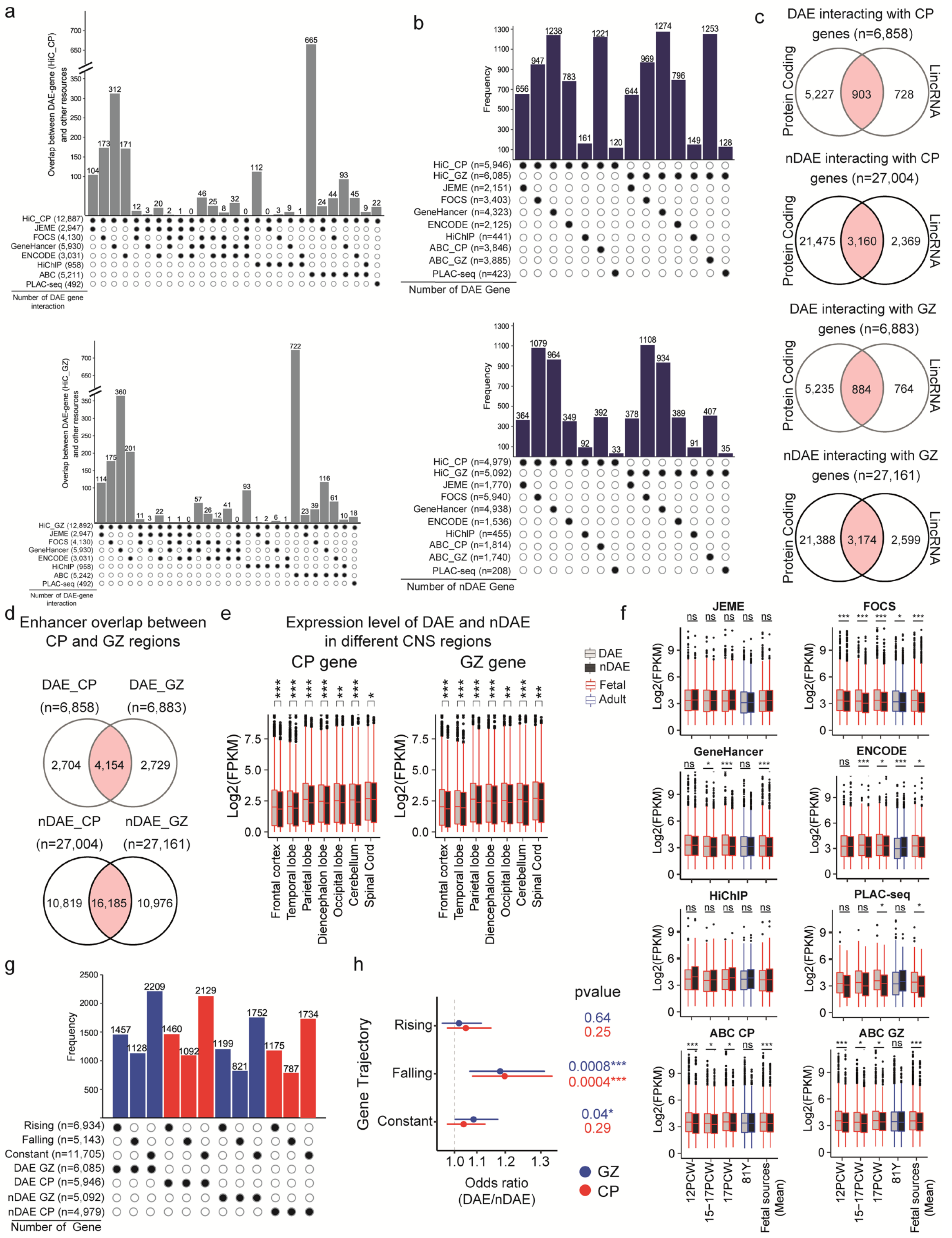


**Fig. S3: Enhancer-Gene predictions and target gene expression**

A) Bar chart showing the overlap between predicted enhancer-gene interactions from HiC of CP (upper panel) or GZ (lower panel) and the alternatively used enhancer-prediction

methods JEME [2], ENCODE [3], FOCS [4],GeneHancer [5], HiChIP [6], PLAC-seq [7] and ABC model [8].

B) Bar chart showing the number of target genes that overlap between the HiC enhancer-gene interaction predictions and the target gene predictions from the alternative methods, for DAEs (upper panel) and nDAEs (lower panel).

C) Venn diagrams showing the interactions of DAEs (first and third panel) or nDAEs (second and fourth panel) with protein coding genes (left) and lincRNA (right) within the same TAD, for interactions from HiC in CP (first and second panel) or GZ (third and fourth panel).

D) Venn diagrams showing the overlap between DAEs (upper panel) or nDAEs (lower panel) that interact with genes in CP (left) or GZ (right).

E) Box plots showing the RNA-seq gene expression levels (in log2 FPKM) of genes linked to DAEs or nDAEs in CP (left) or GZ (right) for different brain regions. Boxes are interquartile range (IQR); line is median; and whiskers extend to 1.5 the IQR. * $p<0.05$; ** $p<0.01$; *** $p<0.001$; (wilcox.test). RNA-seq data obtained from ENCODE project [9].

F) Box plots showing gene expression levels as determined by RNA-seq, for genes that interact with DAEs (light gray) or nDAEs (dark gray) as predicted by JEME [2], FOCS [4], GeneHancer [5], ENCODE [3], HiChIP [6], PLAC-seq [7], or the activity-by-contact (ABC) method [8], as indicated, for either CP or GZ, for fetal (red) or adult (blue) brain samples. Boxes are interquartile range (IQR); line is median; and whiskers extend to 1.5 the IQR. PCW, postconceptional week. FPKM, fragments per kilobase of transcript per million mapped reads. * $p<0.05$; *** $p<0.001$; ns, not significant (wilcox.test). Data obtained from: 12 PCW, Yan et al [10]; 15-17 PCW, De la Torre-Ubieta et al [11]; 17 PCW, Roadmap [12]; 81 years, Roadmap [12]; mean of fetal sources is the mean expression of the first three fetal samples.

G) Bar plot showing the overlap between rising, falling and constantly expressed genes from BrainVar [13] and DAE and nDAE target genes as predicted by HiC in CP or GZ.

H) Line plot showing the odds ratio between DAE and nDAE linked genes in CP (red) or GZ (blue) (as determined by HiC), for rising, falling or constant genes from BrainVar [13]. * $p<0.05$; *** $p<0.001$, Fisher’s exact test.

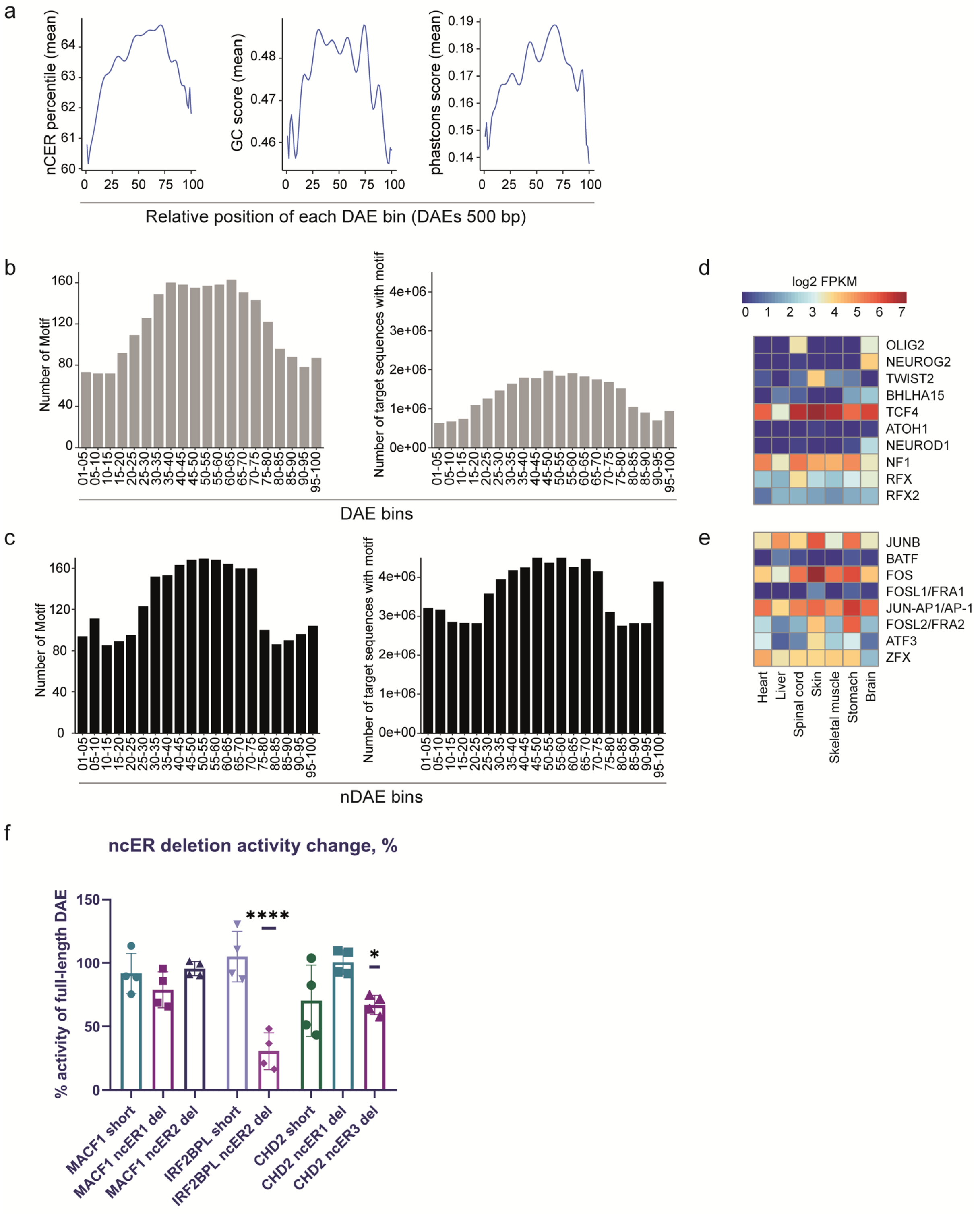


**Fig. S4**: **Features and motifs in DAEs and nDAEs**

A) Line plot showing the distribution of the mean ncER percentile (left) [14], GC content score (middle) [15] and phastcons score (right) [15] over all DAEs that have a size of 500 bp (n= 768).

B) Bar chart showing the number of significant motifs from HOMER analysis (left) or the total number of target sequences for these motifs, across the 20 relative bin groups for DAEs.

C) As B), but now for nDAEs.

D) Heatmap showing the RNA-seq expression levels (Log2 FPKM) of the most enriched TFs at the center of DAEs from the HOMER analysis presented in Fig. 3G, across various human fetal tissues. RNA-seq data obtained from ENCODE project [9].

E) As D), but now for the most enriched TFs at the center of nDAEs from the HOMER analysis reported in Fig. 3H.

F) Effect of ncER deletion on activity of DAEs linked to *IRF2BPL*, *CHD2* and *MACF1*. Percentage of activity of modified DAEs (see methods) compared to the full-length DAE in STARR-seq enhancer reporter experiments is plotted. Two independent transfection experiments were performed, each in duplicate. All data points and standard deviation are shown. * $p<0.05$; **** $p<0.0001$ (one-way ANOVA test followed by multiple comparison test (Fisher's LSD test).

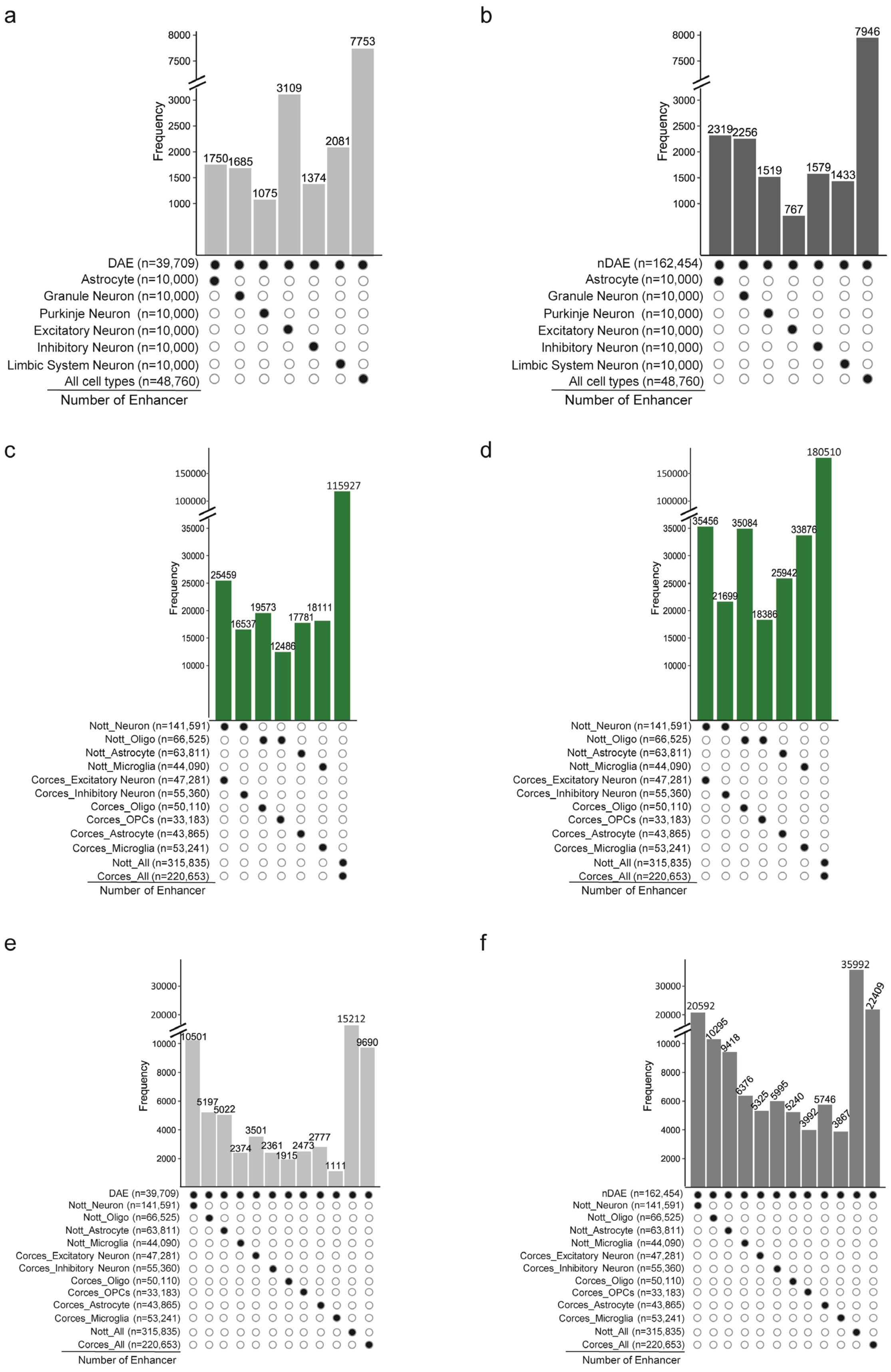


**Fig. S5: Cell type specificity of DAEs and nDAEs**

A) Bar chart showing the overlap between DAEs and cell type-specific chromatin accessibility peaks derived from Domcke et al [16], generated by scATAC-seq on fetal brain.
B) As A), but not nDAEs.
C) Bar chart showing the overlap between cell type specific putative enhancers from postnatal brain from Nott et al [7] and Corces et al [6], using the putative enhancers from Nott et al as reference for the intersection.
D) As C), but now using the putative enhancers from Corces et al as reference for the intersection.
E) Bar chart showing the overlap between DAEs and the postnatal, cell type specific putative enhancers from Nott et al and Corces et al.
F) As E), but now for nDAEs.

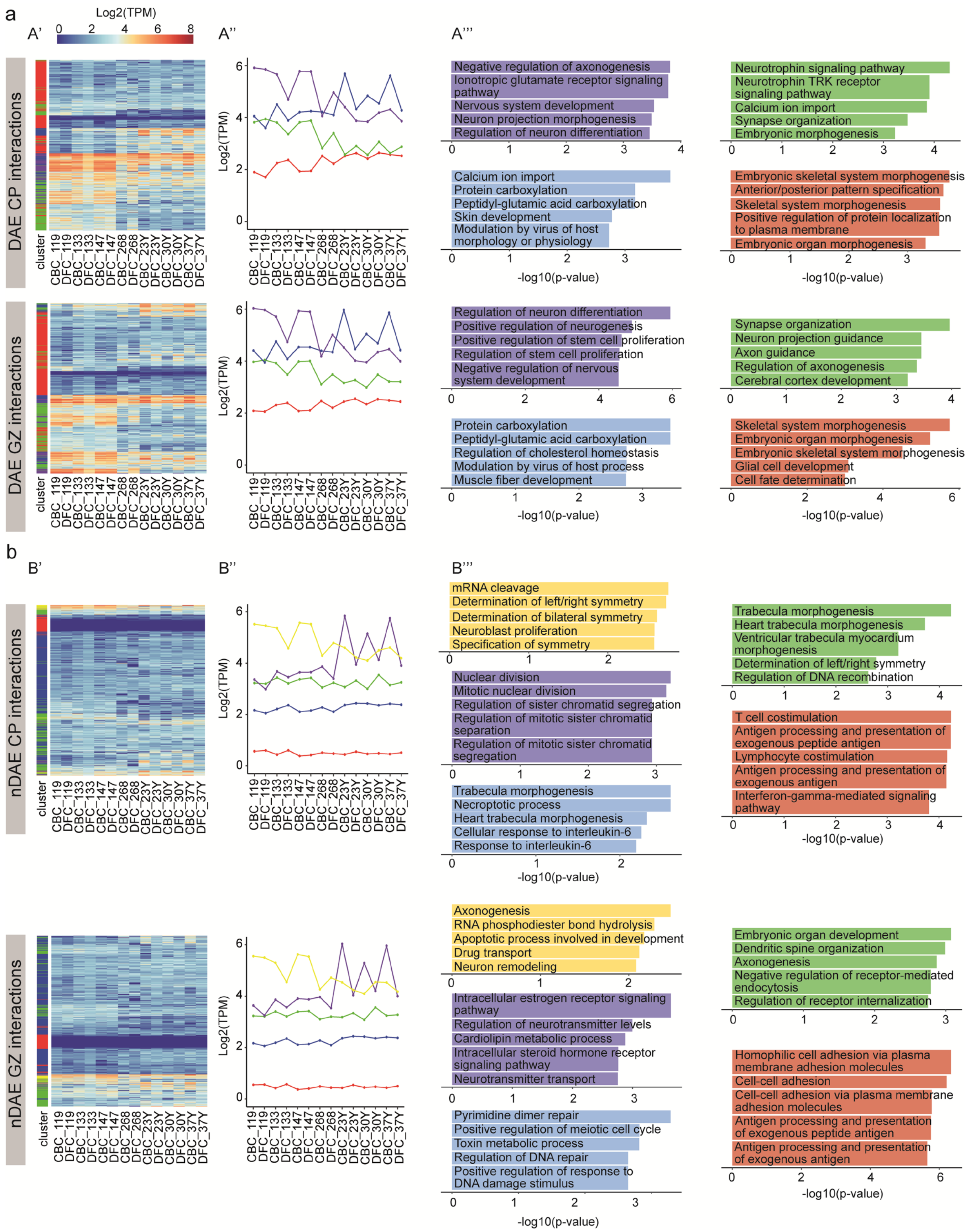


**Fig. S6: Dynamics of DAEs and nDAEs in comparison to adult brain**

A) Heatmap displaying H3K27ac for pre- and postnatal samples from Li et al [17], across all DAEs interacting with protein coding genes in CP (upper heatmap) and GZ (lower

heatmap) ($A^{I}$). K means clustering analysis of H3K27ac enrichment ($A^{II}$) identifies four clusters, depicted in purple, blue, green and red. Level of enrichment is indicated on the y-axis in Log2 TPM. Gene enrichment analysis for the corresponding genes in each cluster ($A^{III}$). X-axis shows the - log 10 (p-value) from Enrichr.

B) As A), but then for nDAEs. K means clustering identifies 5 different clusters for nDAEs, depicted in yellow, purple, green, blue and red.

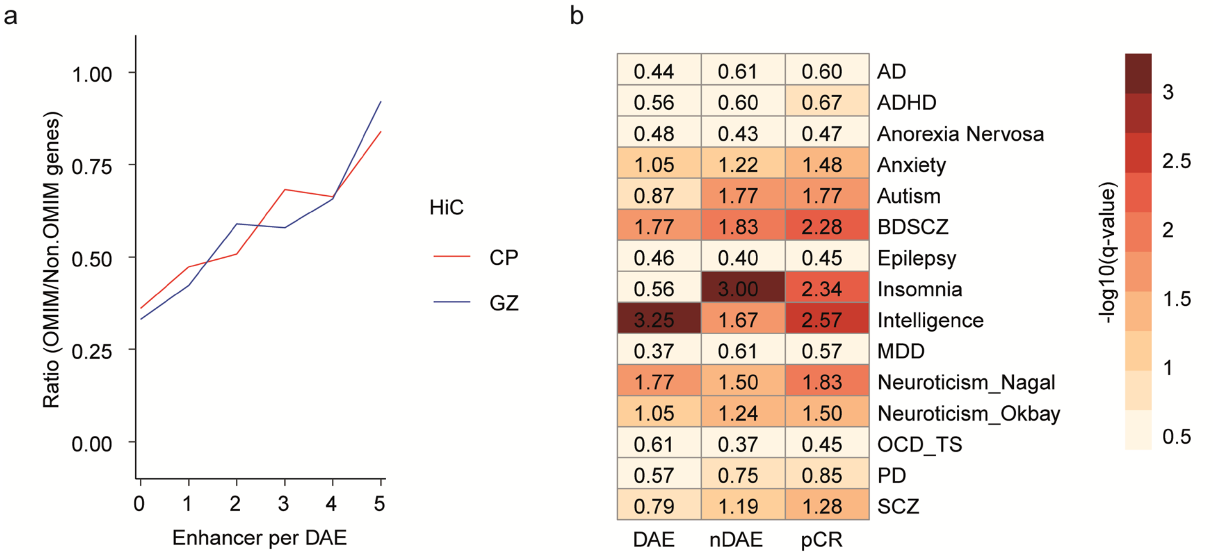


| | DAE | nDAE | pCR |
|---|---|---|---|
| AD | 0.44 | 0.61 | 0.60 |
| ADHD | 0.56 | 0.60 | 0.67 |
| Anorexia Nervosa | 0.48 | 0.43 | 0.47 |
| Anxiety | 1.05 | 1.22 | 1.48 |
| Autism | 0.87 | 1.77 | 1.77 |
| BDSCZ | 1.77 | 1.83 | 2.28 |
| Epilepsy | 0.46 | 0.40 | 0.45 |
| Insomnia | 0.56 | 3.00 | 2.34 |
| Intelligence | 3.25 | 1.67 | 2.57 |
| MDD | 0.37 | 0.61 | 0.57 |
| Neuroticism_Nagal | 1.77 | 1.50 | 1.83 |
| Neuroticism_Okbay | 1.05 | 1.24 | 1.50 |
| OCD_TS | 0.61 | 0.37 | 0.45 |
| PD | 0.57 | 0.75 | 0.85 |
| SCZ | 0.79 | 1.19 | 1.28 |

**Fig. S7: DAEs and nDAEs in human disease, related to Fig. 5**

A) Line graph showing the fraction between OMIM divided by nonOMIM genes as a function of the number of enhancers that a DAE is interacting with, for interactions in CP (red) and GZ (blue). The more enhancers a DAE is interacting with, the more likely it is that the target gene of that DAE is a OMIM gene.

B) Heat map showing the –log10 p-value obtained from LD score regression analysis using relevant publicly available GWAS data for several brain related disorders (see **Additional File 12: Table S11**), for DAEs, nDAEs and pCRs. AD, Alzheimer's disease; ADHD, attention-deficit hyperactivity disorder; BDSCZ, bipolar disorder and schizophrenia; MDD, major depressive disorder; OCD_TS, obsessive compulsive disorder / Tourette syndrome; PD, Parkinson's disease; SCZ, schizophrenia.

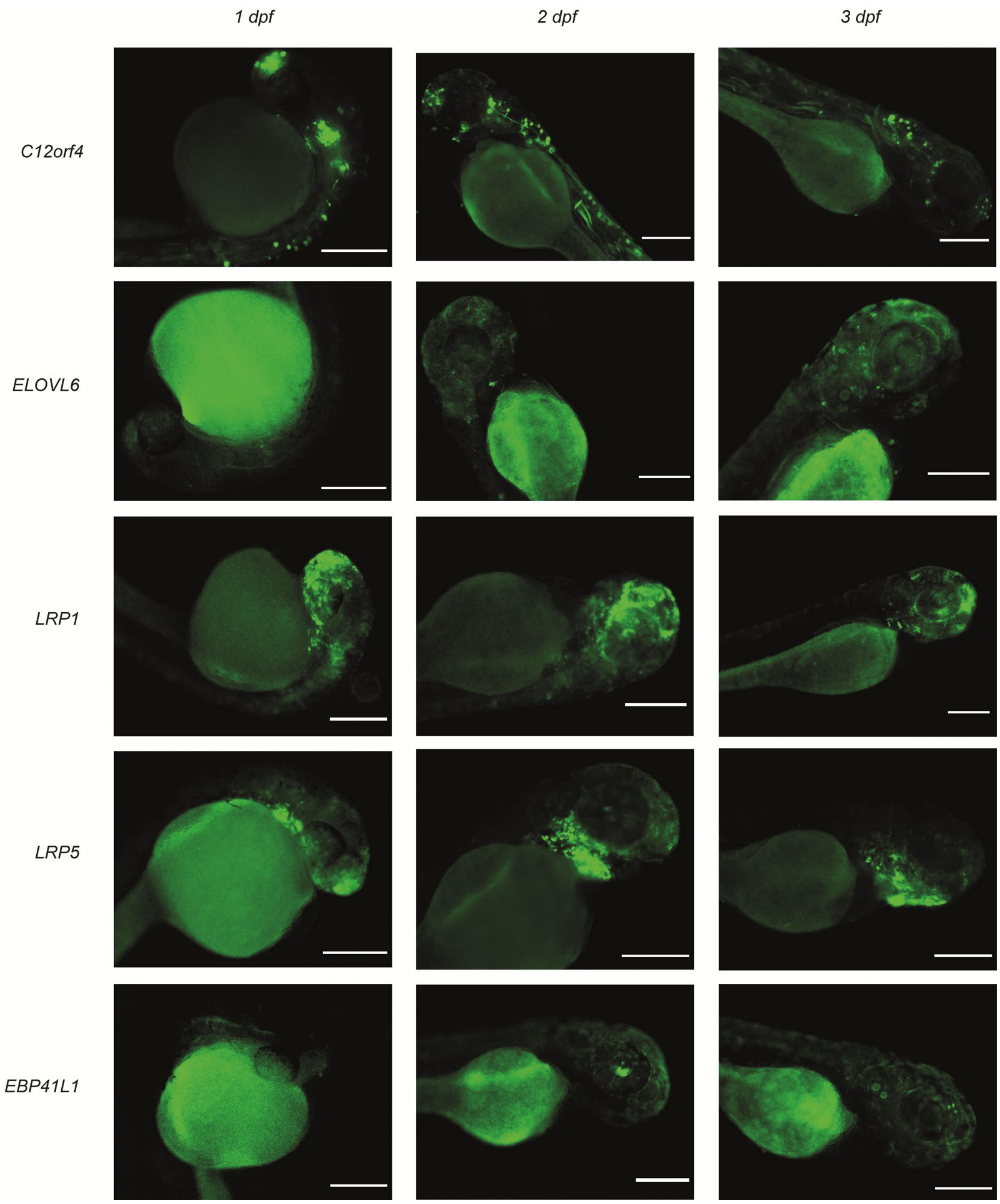


**Fig. S8: Zebrafish enhancer reporter assay**

Panel of additional fluorescent images for validated enhancers, showing GFP expression in zebrafish at 1, 2 and 3 dpf. Scale bars represent 500 µm.

## References


1. McLean CY, Bristor D, Hiller M, Clarke SL, Schaar BT, Lowe CB, Wenger AM, Bejerano G: **GREAT improves functional interpretation of cis-regulatory regions.** *Nat Biotechnol* 2010, **28:**495-501.
2. Cao Q, Anyansi C, Hu X, Xu L, Xiong L, Tang W, Mok MTS, Cheng C, Fan X, Gerstein M, et al: **Reconstruction of enhancer-target networks in 935 samples of human primary cells, tissues and cell lines.** *Nat Genet* 2017, **49:**1428-1436.
3. Ernst J, Kheradpour P, Mikkelsen TS, Shoresh N, Ward LD, Epstein CB, Zhang X, Wang L, Issner R, Coyne M, et al: **Mapping and analysis of chromatin state dynamics in nine human cell types.** *Nature* 2011, **473:**43-49.
4. Hait TA, Amar D, Shamir R, Elkon R: **FOCS: a novel method for analyzing enhancer and gene activity patterns infers an extensive enhancer-promoter map.** *Genome Biol* 2018, **19:**56.
5. Fishilevich S, Nudel R, Rappaport N, Hadar R, Plaschkes I, Iny Stein T, Rosen N, Kohn A, Twik M, Safran M, et al: **GeneHancer: genome-wide integration of enhancers and target genes in GeneCards.** *Database (Oxford)* 2017, **2017**.
6. Corces MR, Shcherbina A, Kundu S, Gloudemans MJ, Fresard L, Granja JM, Louie BH, Eulalio T, Shams S, Bagdatli ST, et al: **Single-cell epigenomic analyses implicate candidate causal variants at inherited risk loci for Alzheimer's and Parkinson's diseases.** *Nat Genet* 2020, **52:**1158-1168.
7. Nott A, Holtman IR, Coufal NG, Schlachetzki JCM, Yu M, Hu R, Han CZ, Pena M, Xiao J, Wu Y, et al: **Brain cell type-specific enhancer-promoter interactome maps and disease-risk association.** *Science* 2019, **366:**1134-1139.
8. Fulco CP, Nasser J, Jones TR, Munson G, Bergman DT, Subramanian V, Grossman SR, Anyoha R, Doughty BR, Patwardhan TA, et al: **Activity-by-contact model of enhancer-promoter regulation from thousands of CRISPR perturbations.** *Nat Genet* 2019, **51:**1664-1669.
9. Consortium EP: **An integrated encyclopedia of DNA elements in the human genome.** *Nature* 2012, **489:**57-74.
10. Yan L, Guo H, Hu B, Li R, Yong J, Zhao Y, Zhi X, Fan X, Guo F, Wang X, et al: **Epigenomic Landscape of Human Fetal Brain, Heart, and Liver.** *J Biol Chem* 2016, **291:**4386-4398.
11. de la Torre-Ubieta L, Stein JL, Won H, Opland CK, Liang D, Lu D, Geschwind DH: **The Dynamic Landscape of Open Chromatin during Human Cortical Neurogenesis.** *Cell* 2018, **172:**289-304 e218.
12. Bernstein BE, Stamatoyannopoulos JA, Costello JF, Ren B, Milosavljevic A, Meissner A, Kellis M, Marra MA, Beaudet AL, Ecker JR, et al: **The NIH Roadmap Epigenomics Mapping Consortium.** *Nat Biotechnol* 2010, **28:**1045-1048.
13. Werling DM, Pochareddy S, Choi J, An JY, Sheppard B, Peng M, Li Z, Dastmalchi C, Santpere G, Sousa AMM, et al: **Whole-Genome and RNA Sequencing Reveal Variation and Transcriptomic Coordination in the Developing Human Prefrontal Cortex.** *Cell Rep* 2020, **31:**107489.
14. Wells A, Heckerman D, Torkamani A, Yin L, Sebat J, Ren B, Telenti A, di Iulio J: **Ranking of non-coding pathogenic variants and putative essential regions of the human genome.** *Nat Commun* 2019, **10:**5241.
15. Siepel A, Bejerano G, Pedersen JS, Hinrichs AS, Hou M, Rosenbloom K, Clawson H, Spieth J, Hillier LW, Richards S, et al: **Evolutionarily conserved elements in vertebrate, insect, worm, and yeast genomes.** *Genome Res* 2005, **15:**1034-1050.
16. Domcke S, Hill AJ, Daza RM, Cao J, O'Day DR, Pliner HA, Aldinger KA, Pokholok D, Zhang F, Milbank JH, et al: **A human cell atlas of fetal chromatin accessibility.** *Science* 2020, **370**.
17. Li M, Santpere G, Imamura Kawasawa Y, Evgrafov OV, Gulden FO, Pochareddy S, Sunkin SM, Li Z, Shin Y, Zhu Y, et al: **Integrative functional genomic analysis of human brain development and neuropsychiatric risks.** *Science* 2018, **362**.
